# FGF10 mitigates doxorubicin-induced myocardial toxicity in mice via activation of FGFR2b/PHLDA1/AKT axis

**DOI:** 10.1038/s41401-023-01101-x

**Published:** 2023-05-24

**Authors:** De-pu Zhou, Lian-cheng Deng, Xiao Feng, Hui-jing Xu, Ye Tian, Wei-wei Yang, Ping-ping Zeng, Li-hui Zou, Xi-hua Yan, Xia-yan Zhu, Dan-hua Shu, Qiang Guo, Xiao-ying Huang, Saverio Bellusci, Zhenkun Lou, Xiao-kun Li, Jin-San Zhang

**Affiliations:** 1 Medical Research Center and the Department of Pulmonary Medicine, The First Affiliated Hospital of Wenzhou Medical University, Wenzhou, 325000 China; 2 International Collaborative Center on Growth Factor Research, School of Pharmaceutical Sciences, Wenzhou Medical University, Wenzhou, 325000 China; 3 Cardio-Pulmonary Institute and Department of Pulmonary and Critical Care Medicine and Infectious Diseases, Universities of Giessen and Marburg Lung Center (UGMLC), Member of the German Center for Lung Research (DZL), Justus-Liebig University Giessen, Giessen, 35392 Germany; 4 Department of Molecular Pharmacology and Experimental Therapeutics, Mayo Clinic, Rochester, MN 55905 USA

**Keywords:** doxorubicin cardiotoxicity, FGF10, FGFR2b, PHLDA1, oxidative stress, DNA damage

## Abstract

Doxorubicin is a common chemotherapeutic agent in clinic, but myocardial toxicity limits its use. Fibroblast growth factor (FGF) 10, a multifunctional paracrine growth factor, plays diverse roles in embryonic and postnatal heart development as well as in cardiac regeneration and repair. In this study we investigated the role of FGF10 as a potential modulator of doxorubicin-induced cardiac cytotoxicity and the underlying molecular mechanisms. *Fgf10*^*+/−*^ mice and an inducible dominant negative FGFR2b transgenic mouse model (*Rosa26*^*rtTA*^*; tet(O)sFgfr2b*) were used to determine the effect of *Fgf10* hypomorph or blocking of endogenous FGFR2b ligands activity on doxorubicin-induced myocardial injury. Acute myocardial injury was induced by a single injection of doxorubicin (25 mg/kg, i.p.). Then cardiac function was evaluated using echocardiography, and DNA damage, oxidative stress and apoptosis in cardiac tissue were assessed. We showed that doxorubicin treatment markedly decreased the expression of FGFR2b ligands including FGF10 in cardiac tissue of wild type mice, whereas *Fgf10*^*+/−*^ mice exhibited a greater degree of oxidative stress, DNA damage and apoptosis as compared with the *Fgf10*^*+/+*^ control. Pre-treatment with recombinant FGF10 protein significantly attenuated doxorubicin-induced oxidative stress, DNA damage and apoptosis both in doxorubicin-treated mice and in doxorubicin-treated HL-1 cells and NRCMs. We demonstrated that FGF10 protected against doxorubicin-induced myocardial toxicity via activation of FGFR2/Pleckstrin homology-like domain family A member 1 (PHLDA1)/Akt axis. Overall, our results unveil a potent protective effect of FGF10 against doxorubicin-induced myocardial injury and identify FGFR2b/PHLDA1/Akt axis as a potential therapeutic target for patients receiving doxorubicin treatment.

## Introduction

For decades, doxorubicin (DOXO) has been the first-line chemotherapeutic treatment for various types of malignancies, such as bladder cancer, breast cancer, leukemia, and lymphoma [1, 2]. The combined use of DOXO with other chemotherapies is a mainstream treatment strategy for a broad range of tumors [3]. However, the clinical utilization of DOXO is limited by its dose-dependent side effects, among which DOXO-induced cardiomyopathy leading to congestive heart failure is the most lethal one [4, 5]. DOXO-induced myocardial cytotoxicity is underpinned by several mechanisms. The first is oxidative stress. DOXO is converted into an unstable intermediate, which is quickly converted back into DOXO, generating free radicals in cells [6]. Another mechanism is via DOXO intercalation into cellular DNA, which induces double-strand breaks (DSBs). DOXO targets Topoisomerase-II (Top2) to form a Top2β-DOXO-DNA ternary cleavage complex, which induced DSBs in adult cardiomyocytes [7]. The accumulation of oxidative free radicals and DSBs further promotes the apoptosis cascade, induces cardiotoxicity, and ultimately results in myocardial injury [8]. Dexrazoxane is the only drug approved by US FDA to prevent DOXO induced cardiotoxicity in cancer patients. It is used in conjunction with the anthracycline to decrease the incidence of cardiomyopathy and heart failure [9]. It is generally believed that dexrazoxane counteracts DOXO toxicity by removing iron from iron/DOXO complexes and also inhibiting Top2 activity [10]. However it also has side effects, including myelosuppression and increased risk of secondary malignancy [11]. Therefore, it is crucial to better understand the pathological mechanisms and develop novel strategies to ameliorate DOXO’s cardiotoxicity.

Fibroblast growth factor (FGF) 10, a member of the FGF7 subfamily that signals through the binding of and activation of its specific receptors (FGFR1b and FGFR2b), plays an essential role in organogenesis during embryonic development and also contributes to maintenance of adult homeostasis. Recent studies from different laboratories including ours highlight a role of FGF10 in protection against various injuries, such as renal and hepatic ischemic/reperfusion [12, 13], neonatal hypoxic-ischemic brain injury [14], bleomycin and particulate matter-induced lung injury [15–17].

Several studies have shown that FGF10 also plays an important role in embryonic and postnatal heart development, as well as adult cardiac tissue repair and regeneration [18]. FGF10 is a specific marker for the second heart field, which contains progenitor cells and gives rise to the atria and ventricles [19], and regulates fetal cardiomyocyte proliferation. FGF10 is necessary for neonatal development and is essential for cardiomyocyte regeneration [20]. FGF10 overexpression at around postnatal day 7 upregulates epicardial cell expansion, which is beneficial for cardiac repair after injury [21]. Other studies have demonstrated the protective function of FGF10 in cardiac diseases. Wang et al. identified the protective role of the heparin-based coacervate of FGF10 in acute myocardial infarction [22]. Rochais lab has previously shown that FGF10 promotes cell cycle re-entry in adult cardiomyocytes [20]. More recent study further demonstrates that FGF10 overexpression alleviates myocardial injury response by pushing the mitosis-refractory myocardium into a more proliferative state, whereas FGF10 haploinsufficiency disrupts this process, which causes further pathological remodeling and decline in cardiac function [23]. Collectively, the evidence suggests that FGF10 could be a potentially useful cardioprotective agent. However, no studies have investigated the effect of FGF10 in DOXO-induced cardiotoxicity.

The current study aimed to investigate the role and the molecular underpinnings of FGF10 signaling in DOXO-induced cardiotoxicity. Both FGF10 protein and transgenic mice, including *Fgf10*^*+/−*^ and an inducible dominant negative FGFR2b transgenic model, were employed to determine how exogenous FGF10 and genetic manipulations of endogenous FGF10 and FGFR2b activity may impact DOXO-induced myocardial injury. The findings reveal that endogenous FGF10/FGFR2b signaling is intimately involved in DOXO-induced cardiac cytotoxicity, and administration of exogenous FGF10 protein is capable of significantly mitigating the myocardial lesion caused by DOXO through regulation of PHLDA1/Akt signaling axis.

## Materials and methods

### Breeding, genotyping and maintenance of animals and drug administration in vivo

Protocols involving animals were approved by Wenzhou Medical University Committee on Ethics in the Care and Use of Laboratory Animals(Ethics Approval Number: 2021-236). Wild-type (WT) male C57BL/6 mice were purchased from the Laboratory Animal Center of Shanghai (Shanghai, China). The *Fgf10*^*+/−*^ mice were kindly provided by Prof. Nan Tang (National Institute of Biological Sciences, Beijing, China). The *Rosa26*^*rtTA/rtTA*^ and *tet(o) sFgfr2b/+* mice were acquired from Bellusci Lab (Giessen University, Germany). *Fgf10*^*+/−*^ mice, *Rosa26*^*rtTA/rtTA*^ and *tet(o) sFgfr2b/+* mice have previously been described [24, 25]. The experimental mice for inducible expression of dominant negative FGFR2b (*Rosa26*^*rtTA*/*rtTA*^*; tet(o)sFgfr2b/*+) littermate control (*Rosa26*^*rtTA*/*rtTA*^*;* +*/*+) were generated by crossing *Rosa26*^*rtTA*/*rtTA*^*; tet(o)sFgfr2b/*+ and *Rosa26*^*rtTA*/*rtTA*^*;* +/+ animals. All transgenic mice were genotyped at postnatal day 14 using the mouse Direct PCR Kit (Biomake, Houston, TX, USA). Genomic DNA was extracted from tails and prepared for PCR amplification. PCR primers for *rtTA*, *Fgf10* and *Fgfr2b* are listed in Supplementary Table S1. PCR products were separated by agarose gel electrophoresis with a representative genotyping result shown in Supplementary Fig. S1a, b.

All mice were housed in the experimental animal center of the Wenzhou Medical University in a temperature-controlled facility with a 12 h light/dark cycle, and allowed access to food and water *ad libitum*. They received human care according to the “Guide for the Care and Use of Laboratory Animals” prepared by the National Academy of Sciences and published by the National Institutes of Health (NIH publication 86-23 revised 1985).

The experimental setups are depicted in Supplementary Fig. S2a, c, d. After one week of adaptive feeding, a total of eighty C57BL/6J mice of indicated genotypes were divided into four groups: control, FGF10, DOXO, and DOXO + FGF10. Mice in DOXO + FGF10 group and FGF10 groups received intraperitoneal (i.p.) injections of recombinant FGF10 proteins (Wenzhou Medical University, Wenzhou, China) at 5 mg/kg for five consecutive days, in accordance with our previous study [13]. Meanwhile, other groups were i.p. injected with an equal volume of saline. Subsequently, mice in the DOXO and DOXO + FGF10 groups received a single i.p. injection of Doxorubicin-HCl (MCE, New Jersey, USA) at 25 mg/kg to mimic acute DOXO induced myocardial injury demonstrated by previous studies [26]. To investigate the effect of *Fgf10* on DOXO-induced cardiotoxicity, *Fgf10*^*+/-*^ and *Fgf10*^*+/+*^ mice were i.p. injected with a single dose of DOXO at 25 mg/kg after adaptive feeding. For FGF10 treatment, mice were i.p. injected with a single dose of FGF10 at 5 mg/kg after adaptive feeding. Equal volumes of saline were administered for the controls of both DOXO and FGF10 treatment. There were 20 mice in each group.

In order to inhibit FGFR2b ligands, the double transgenic mice (*DTG*), *Rosa26*^*rtTA/rtTA*^*; tet(O) sFgfr2b/+*, and the control mice (*CTRL*), *Rosa26*^*rtTA/rtTA*^ *+/+*, were divided into four groups with 20 mice/group: *DTG* + DOXO, *DTG* + DOXO + FGF10, *CTRL* + DOXO and *CTRL* + DOXO + FGF10 groups. All mice in these four groups received 20 mg/kg of doxycycline for six consecutive days. From the second day, mice in *DTG* + DOXO + FGF10 and *CTRL* + DOXO + FGF10 groups were treated with FGF10 at 5 mg/kg for 1 h after the doxycycline injection. Meanwhile, the other two groups were injected an equal volume of normal saline as control. On the final day, all groups were i.p. injected with DOXO. Mice were euthanized with isoflurane and the heart tissues were assessed for gross anatomical and histopathological changes, as well as protein expression at day 5 post DOXO injection.

### Echocardiography analysis

Echocardiography analysis was performed 5 days post DOXO injection. Mice were euthanized with isoflurane and transthoracic echocardiography was performed using a Vevo1100 cardiovascular research ultrasound machine (Visualsonics, Ontario, Canada). The left ventricular end‐diastolic diameter (LVEDd) and left ventricular end‐systolic diameter (LVESd) were measured. The left ventricular ejection fraction (LVEF) and left ventricular fractional shortening (LVFS) were measured according to LVEDd and LVESd.

### Measurement of cardiac Troponin I

Blood samples were collected from mice 3 days post DOXO injection to measure plasma cardiac Troponin I (cTnI). In accordance with the instructions, the cTnI ELISA kit (Jianglaibio, Shanghai, China) was used to detect acute cardiac injury.

### Plasmids

The plasmids for *PHDLA1* shRNA were constructed using *pLKO-1* lentiviral expression vector and the gRNA is listed in Supplementary Table S1. The plasmids for FGFR2 were constructed using *pCDH-CMV-MCS-EF1-CopGFP-T2A-Puro* vector. The FGFR2 kinase dead mutant *Y657/658F* plasmid and a constitutively active FGFR2 mutant *C383R* plasmid were designed and constructed based on the previously published information [27, 28].

### Neonatal rat cardiomyocyte (NRCM) isolation

The hearts were harvested from 2–3 day-old neonatal rats. They were digested three times for 10 min each by incubating at 37 °C with a protease solution (0.1% collagenase type IV, 0.25% trypsin, 1 U/mL DNase I, 116 mM NaCl, 20 mM HEPES, 12.5 mM NaH_2_PO_4_, 5.6 mM glucose, 5.4 mM KCl, and 0.8 mM MgSO_4_ [pH 7.35]), as described in a previous publication [29]. The isolated cells were collected by centrifugation and incubated on a 10-cm culture plate for 1 h at 37 °C. Unattached cells were collected and seeded on laminin-coated 10-cm cell culture plates.

### Cell culture, lentiviral packaging, transfection and selection of stable cells

HL-1 cells, NRCM and HEK293T cells were cultured in DMEM (Gibco, Massachusetts, USA) containing 10% fetal bovine serum (FBS) (Gibco, Massachusetts, USA) and 1% penicillin and streptomycin (Gibco, Massachusetts, USA). The experimental cell setups are depicted in Supplementary Fig. S2b. HEK293T cells were used for lentiviral packaging. The plasmid *pMD2.5*, *psPAX* and targeted plasmid were transfected into 293T cells by lipo3000 (Thermo Fisher, Massachusetts, USA). After 48 h, the lentivirus-containing medium was harvested. HL-1 cells were infected with the lentivirus along with 8 μg/mL polybrene (Solarbio, Beijing, China) and selected for 72 h with 1 µM puromycin (Invitrogen, Massachusetts, USA).

### 3-(4,5-Dimethylthiazol-2-yl)-2,5-diphenyl tetrazolium bromide assay

The 3-(4,5-dimethylthiazol-2-yl)-2,5-diphenyl tetrazolium bromide (MTT) (Solarbio, Beijing, China) assay was used to determine cell viability. Each well of the 96 well plates was seeded with 2 × 10^5^ cells. After 24 h of culture, cells were treated with DOXO or FGF10 at desired concentrations. After incubation, MTT solution at 5 mg/mL was added to the cells, and they were then incubated in the dark for 4 h at 37 °C. Afterwards, the medium was removed and 100 μL solution of 4% dimethyl sulfoxide (DMSO) (Solarbio, Beijing, China) was added to cells before measuring the absorbance at 570 nm.

### Drug treatment of cultured cells

HL-1 and NRCM cells were serum-starved overnight in DMEM with 0.1% FBS prior to use. The cells were divided into four groups: CONTROL, FGF10, DOXO, and DOXO + FGF10. The FGF10 and DOXO + FGF10 groups were pretreated with FGF10 at a concentration of 100 ng/mL, while control and DOXO groups received same volume of phosphate buffered saline (PBS). After 30 min, the DOXO and DOXO + FGF10 groups were treated with 0.5 µM DOXO for 24 h. The other groups received the same volume of PBS. To block the Akt pathway, HL-1 cells were treated with 10 nM MK2206 (MCE, New Jersey, USA) for 30 min before FGF10.

### Western blotting (WB)

Proteins were extracted from cells or heart tissues using a RIPA lysis buffer (Beyotime, China) freshly supplemented with protease and phosphatase inhibitors (Boster, China). Bradford assay was used to determine the protein concentrations in the samples (Bio-Rad, USA). Subsequently, the protein samples were electrophoresed in sodium dodecyl sulfate-polyacrylamide (SDS-PAGE) gel and transferred to PVDF membranes (Millipore, Massachusetts, USA). Membranes were blocked with 5% bovine serum albumin (BSA) (Solarbio, Beijing, China) for 2 h at room temperature. The membranes were incubated with primary antibodies overnight at 4 °C. The primary antibodies are listed in Supplementary Table S2. Afterwards, the membrane was incubated with the secondary antibodies for 1 h at room temperature and visualized by the ECL reagent. The membranes were detected using ChemiDoc™ XRS + system and analyzed using an Image Lab software (Bio-Rad, California, USA). The intensity values of the relative protein levels were normalized to GAPDH.

### Immunohistochemistry (IHC) and Hematoxylin-eosin stain (H&E)

Heart tissues were fixed in 4% paraformaldehyde for 24 h at 4 °C. The tissues were then embedded in paraffin, cut into 5 μm sections in thickness, and placed on glass slides. The paraffin slides were dewaxed, rehydrated, and stained with H&E Kit (Solarbio, Beijing, China). For the IHC staining, the paraffin slides were dewaxed, rehydrated, and subjected to antigen retrieval in sodium citrate buffer (pH 6.0) or Tris-EDTA buffer (pH 9.0). IHC was performed using a streptavidin-peroxidase kit (ZSGB-BIO, Beijing, China) according to the manufacturer’s instructions. After visualization with a DAB kit (Solarbio, Beijing, China), the slides were counterstained with hematoxylin and sealed with neutral resin.

### Immunofluorescence (IF)

Cardiac tissue was snap-frozen in liquid nitrogen, embedded in OCT and cut into 10 µm sections in the cryostat. The slides were then rewarmed at room temperature for an hour. After blocking with 5% BSA for 2 h at room temperature, the slides were incubated at 4 °C overnight with the indicated primary antibodies (listed in Supplementary Table S2). After washing, the slides were incubated with fluorescent secondary antibody (ThermoFisher, Massachusetts, USA) for 2 h at room temperature. Finally, the sections were covered with ProLong Antifade Mountant with DAPI (ThermoFisher, Massachusetts, USA).

### RNA extraction and quantitative real-time PCR (qRT-PCR)

Total RNA extraction was carried out using an RNAiso Plus reagent (TaKaRa, Kyoto, Japan). cDNA was synthesized using RevertAid™ Master Mix (ThermoFisher, USA). Gene expression analysis was performed using the SYBR Premix Ex Taq (TaKaRa, Kyoto, Japan) and performed in CFX96 Real-Time System (Bio-Rad, California, USA). As an internal control, the results were normalized to *ACTB* mRNA.

### Superoxide dismutase (SOD) activity, malondialdehyde (MDA) and lactate dehydrogenase (LDH) release detection

The quantitative measurement of SOD activity, MDA and LDH release were carried out in accordance with the manufacturer’s instructions (Beyotime, Shanghai, China).

### TUNEL staining

Apoptosis was detected in paraffin slides using a terminal deoxynucleotidyl transferase-mediated nick-end labeling (TUNEL) assay kit based on the manufacturer’s instruction (Vazyme, Nanjing, China). The nucleus was labeled with DAPI.

### Neutral comet assay

The neutral comet assay was used to assess for double-stranded DNA breaks. Mice were sacrificed at 5 days after DOXO injection, while NRCM and HL-1 cells were harvested at 24 h after DOXO administration. Cardiac tissues from each group were digested with collagenase I and DNAase to single cell suspensions and HL-1 cells were digested by trypsin-EDTA. Single cells were resuspended in 100 µL of 0.75% low melting point agarose (Solarbio, Beijing, China). The suspension was applied to the slides covered with a layer of 1% normal melting point agarose (Solarbio, Beijing, China). The slides were then submersed in lytic solution at 4 °C overnight (2.5 M NaCl, 0.1 M EDTA, 10 mM Tris-base, 1% N-laurylsarcosine, 0.5% Triton X-100, 10% dimethyl sulfoxide). After lysing, the slides were balanced for 15 min in electrophoresis buffer (300 mM sodium acetate, 300 mM sodium acetate) at 4 °C. Afterward, the slides were placed in a horizontal electrophoresis chamber at 18 V for 1 h. Slides were washed in PBS before being fixed in ethanol and dried at room temperature. Finally, slides were stained with propidium iodide.

### DCFH-DA, DHE, JC-1 and Mito-Tracker staining

Reactive oxygen species (ROS) generation was measured using dihydroethidium (DHE) and dihydrodichlorofluorescein diacetate (DCFH-DA) staining. JC-1 and Mito-Tracker were utilized to detect mitochondria changes. HL-1 cells were harvested at 24 h post DOXO administration and incubated with DCFH-DA, JC-1, Mito-Tracker and DHE in accordance with the manufacturer’s instructions (Beyotime, Shanghai, China). The fluorescence intensities of DCFH-DA and DHE were measured by flow cytometry. The JC-1 and Mito-Tracker were observed under a fluorescence microscope.

### Co-immunoprecipitation (Co-IP)

HEK293T cells were transfected with *FGFR2-C383R* and *PHLDA1-HA* expression vectors using Lipo3000 transfection regent according to manufacturer’s instructions. Cells were harvested at 48 h after transfection. They were then washed with PBS and lysed with NP40 lysis buffer (Beyotime, shanghai, China) freshly supplemented with phosphatase inhibitors. Cellular extracts were incubated with the anti HA Tag-agarose conjugated beads overnight at 4 °C. After washing, the IP pulled down protein complexes were subjected to Western blot.

### Statistical analysis

Continuous variables were expressed as mean ± standard error of mean (SEM). For IF and IHC, we selected three mice per group and randomly choose five microscopic field per section (total 15 sections per group). For the comet assay, we randomly choose 50 cells per group. For γ-H2AX and 53BP1 foci number, we randomly chose 75 cells per group. Foci were counted by Focinator [30]. Colocalization in IF was analyzed by JACOP [31]. For Western blot, the gray value of each band was divided by the gray value of the corresponding GAPDH band to obtain the normalized band density, and then normalized to the control group. We used an unpaired *t*-test to compare significance between two groups. One-way ANOVA or Wilcoxon rank sum tests followed by the Tukey post hoc test was used to compare the difference between more than two groups. A *P* value < 0.05 was considered statistically significant.

## Results

### Down-regulation of FGF10 in doxorubicin-induced myocardial injury

**Fig. 1:**
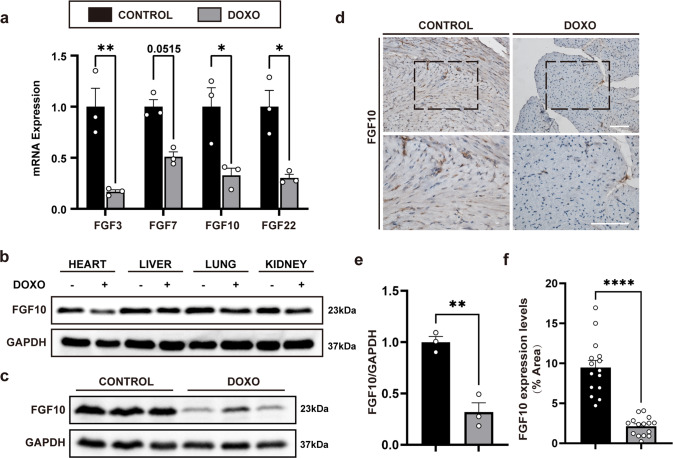
Down regulation of FGF10 in DOXO-induced myocardial injury. **a** Expression of FGF7 subfamily members in doxorubicin-induced myocardial injury determined by qRT-PCR. **b** Western blotting analysis of FGF10 protein expression in major viscera after doxorubicin injection. **c** Western blotting analysis of FGF10 protein expression in the mouse heart after doxorubicin injection. **d** Immunohistochemical staining of FGF10 in cardiac sections. Top row: ×200 magnification. Bottom row: ×400 magnification. **e** Statistical analysis of Western blotting presented in **c**. The results are shown as relative gray value. **f** Statistical analysis of immunohistochemistry in **d**. The results are shown as positive staining area per total area of the heart (% Area). GAPDH was used as an internal control. DOXO doxorubicin, FGF10 Fibroblast growth factor 10. ns non-significant; *P < 0.05; **P < 0.01; ****P < 0.0001. Scaler bar: 100 μm.

Five days after DOXO treatment, the mRNA expression of FGF7 subfamily members in the heart tissues was determined. *Fgf10* mRNA, as well as the remaining FGF7 subfamily members, was significantly decreased (Fig. 1a). DOXO-exposure are known to cause cardiac injury as well as injury to other organs, such as the liver and kidney. Thus, FGF10 expression in several other visceral tissues was also measured by immunoblot 5 days following DOXO treatment (Fig. 1b). We found that FGF10 expression was only significantly lowered in the heart in the DOXO-treated group when compared to the control group (Fig. 1b, c, e), but no difference in FGF10 protein expression in the liver, kidney, or lung between the DOXO-treated group and the control group (Fig. 1b). A similar result was observed with IHC staining for FGF10 in heart (Fig. 1d, f). Therefore, FGF10 expression was only markedly reduced in the heart after DOXO administration.

### Decreased Fgf10 dosage aggravates DOXO-induced myocardial injury and cardiac dysfunction

**Fig. 2:**
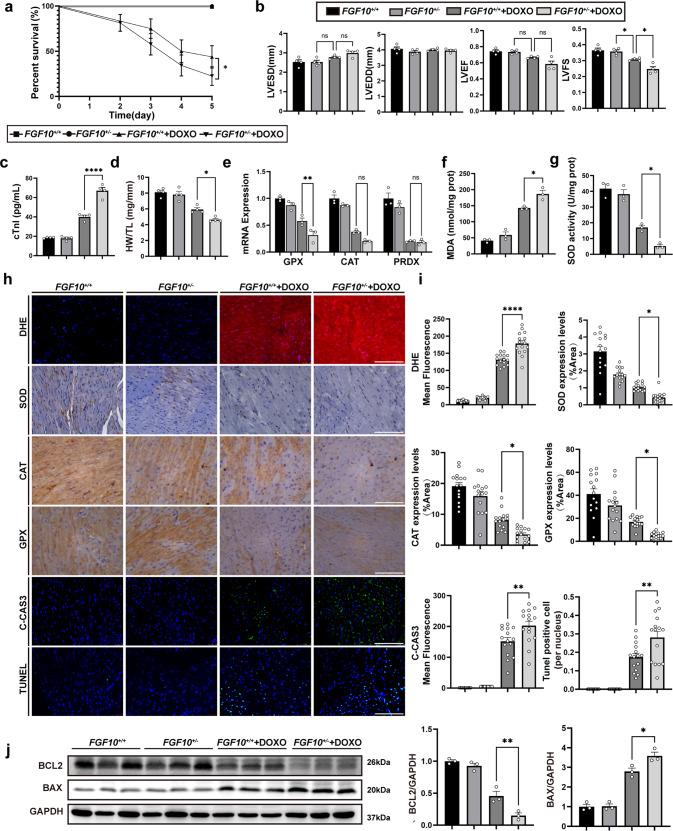
Reducing Fgf10 dosage aggravates DOXO-induced cardiac dysfunction and myocardial injury. **a** Survival rates in each group of mice 5 days after doxorubicin injection. **b** Echocardiographic assessment of mouse cardiac function in each group 5 days after doxorubicin administration. **c** cTnI concentration in each group at day 3 post doxorubicin injection. **d** HW/TL in each group at day 5 post doxorubicin injection. **e** qRT-PCR analysis of antioxidant enzyme gene expression, including *GPX*, *CAT*, and *PRDX*at day 5 post doxorubicin injection. **f**, **g** MDA content and SOD activity in each group at day 5 post doxorubicin injection. **h** Representative immunohistochemistry and immunofluorescence (×400 magnification) images from each group, stained for DHE, SOD, CAT, GPX, C-CAS3, and TUNEL, respectively, as indicated. **i** Statistical analysis of immunohistochemistry and immunofluorescence presented in **h**. The results of immunohistochemistry are shown as positive area per total area (%Area). The results of immunofluorescence are represented as the mean fluorescence intensity. **j** Western blotting and statistical analysis of Bcl-2 and Bax expression in the heart. GAPDH was used as an internal control. ns, non-significant; *P < 0.05; **P < 0.01; ****P < 0.0001. cTnI Cardiac troponin, LVEDd left ventricular end-diastolic diameter, LVESd left ventricular end-systolic diameter, LVEF left ventricular ejection fraction, LVFS left ventricular fractional shortening, HW/TL the ratio of heart weight and tibia length, SOD Superoxide dismutase, PRDX peroxiredoxin, CAT catalase, GPX Glutathione peroxidase, Bcl2 B-cell lymphoma 2, TUNEL TdT-mediated dUTP Nick-End Labeling, BAX BCL2-associated X protein, MDA Malondialdehyde, DHE Dihydroethidium, C-CAS3 Cleaved-Caspase-3. Scale bar: 100 μm.

*Fgf10*^*+/−*^ mice were used to examine the role of FGF10 in DOXO-induced myocardial injury. As expected, the FGF10 protein expression of heart was lower in *Fgf10*^*+/−*^ mice compared to that of *Fgf10*^*+/+*^ mice (Supplementary Fig. S1c, d). Under normal conditions, *Fgf10* knockdown had no effect on oxidative stress and apoptosis, which is consistent with previous study that the cardiac function of *Fgf10*^*+/−*^ mice was indistinguishable from that of control littermates [23]. There were no macroscopic changes found in H&E staining between each group (Supplementary Fig. S3a). The *Fgf10*^*+/−* ^+ DOXO group had a lower survival rate under pathological conditions (Fig. 2a). Meanwhile, compared with the *Fgf10*^*+/+*^ + DOXO group, a reduction in *Fgf10* dosage degraded cardiac function, and LVFS was significantly decreased in the *Fgf10*^*+/−* ^+ DOXO group (Fig. 2b; Supplementary Fig. S3b) suggesting a more severe myocardial injury. After DOXO injection, the *Fgf10*^*+/−* ^+ DOXO group had a higher serum cTnI concentration, which is a specific indicator of myocardial damage, and a lower heart weight to tibia length (HW/TL) ratio (Fig. 2c, d). Given that oxidative stress and apoptosis are the main mechanisms underpinning DOXO-induced myocardial injury, changes in parameters of oxidative stress and apoptosis were measured. After DOXO administration, the *Fgf10*^*+/−* ^+ DOXO group had a higher mean DHE fluorescence intensity as well as higher MDA content, indicating higher levels of oxidative stress (Fig. 2f, h, i). Moreover, the antioxidant response was reduced in the context of *Fgf10* knockdown. Compared with the *Fgf10*^*+/+*^ + DOXO group, the protein expression and activity of SOD were both reduced in the *Fgf10*^*+/−*^ + DOXO group (Fig. 2g, h, i), along with a reduction in the mRNA and protein expression of additional antioxidant enzymes, including glutathione peroxidase (GPX) and catalase (CAT) (Fig. 2e, h, i), but no difference in the mRNA expression of peroxiredoxin1 (PRDX1) between the two groups (Fig. 2e). Similarly, an increase in apoptosis was observed in the *Fgf10*^*+/−*^+DOXO group. The *Fgf10*^*+/−*^ group had a higher mean cleaved caspase 3 (C-CAS3) fluorescence intensity and more TUNEL-positive cells than the *Fgf10*^*+/+*^ + DOXO group (Fig. 2h, i). Increased Bcl-2-associated X protein (Bax) expression and decreased B-cell lymphoma 2 (Bcl-2) protein expression (Fig. 2j) were also indicative of more severe apoptosis. These findings suggest that FGF10 may play a protective role in DOXO-induced myocardial injury. A reduction in FGF10 protein level impaired cardiac function, intensified oxidative stress, and increased apoptosis caused by DOXO.

### Exogeneous FGF10 preserves cardiac function via mitigation of DOXO-induced oxidative stress and apoptosis in vivo

**Fig. 3:**
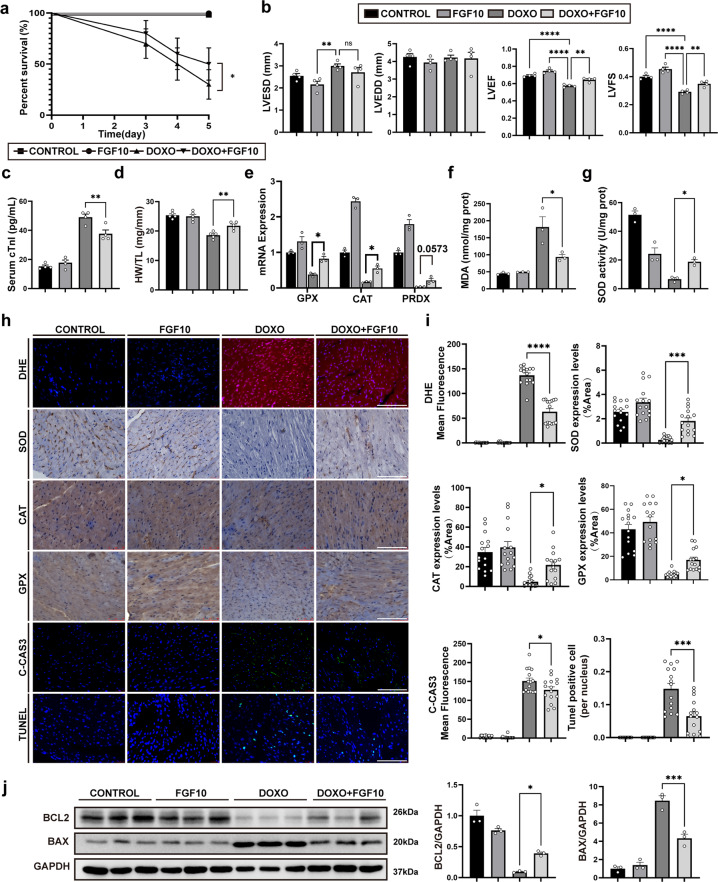
Exogeneous FGF10 mitigates DOXO-induced oxidative stress and apoptosis, and ameliorates cardiac function. **a** Survival rate of each group after 5 days past doxorubicin injection. **b** Echocardiographic assessment of mice in each group 5 days after doxorubicin administration. **c** cTnI concentration in each group at 3 days post doxorubicin injection. **d** HW/TL in each group at day 5 post doxorubicin injection. **e** qRT-PCR analysis of antioxidant enzyme gene expression, including *GPX*, *CAT*, and *PRDX*. **f**, **g** MDA content and SOD activity in each group. **h** Representative immunohistochemistry and immunofluorescence images (×400 magnification) in each group with indicated staining. **i** Statistical analysis of immunohistochemistry and immunofluorescence presented in **h**. The results of immunohistochemistry are shown as positive area per total area (%Area). The results of immunofluorescence are presented as the mean fluorescence intensity. **j** Western blotting and statistical analysis of Bcl-2 and Bax. GAPDH was used as an internal control. ns, non-significant; *P < 0.05; **P < 0.01; ***P < 0.001; ****P < 0.0001. Scaler bar: 100 μm.

To investigate the potential cardioprotective effect of FGF10, we administered FGF10 as a pre-treatment prior to DOXO injection. Doxorubicin administration resulted in loss of cardiac mass as well as impairment of cardiac function. Compared with the DOXO alone group, FGF10 reduced the death rate (Fig. 3a), and preserved cardiac function, alleviated myocardial injury, and increased heart weight (Fig. 3b–d; Supplementary Fig. S3d). In the DOXO + FGF10 group, FGF10 significantly increased the LVFS and HW/TL and demonstrated a lower cTnI concentration when compared to the DOXO group. However, no macroscopic changes were detected based on H&E staining (Supplementary Fig. S3d). We further investigated the effect of FGF10 on DOXO-induced oxidative stress and apoptosis. GPX and CAT mRNA and protein expression were significantly increased, whereas MDA was decreased in the DOXO + FGF10 group compared to the DOXO group (Fig. 3e, f, h, i). The expression of PRDX1 mRNA in the DOXO + FGF10 group appeared to be higher than in the DOXO alone group, but this difference was not statistically significant (Fig. 3e). The protein expression and enzyme activity of SOD were upregulated following FGF10 treatment (Fig. 3g–i). Treatment with FGF10 also attenuated the apoptotic cell death after DOXO injection as indicated by reduced mean fluorescence intensity of C-CAS3 and the number of TUNEL-positive cells (Fig. 3h, i). Consistently, immunoblot demonstrated a decrease in Bax and an increase in Bcl-2 in the DOXO + FGF10 group (Fig. 3j). These results together indicate that FGF10 protects against doxorubicin-induced myocardial injury in vivo.

### Exogeneous FGF10 mitigates DOXO-induced cardiomyocyte cytotoxicity in vitro

**Fig. 4:**
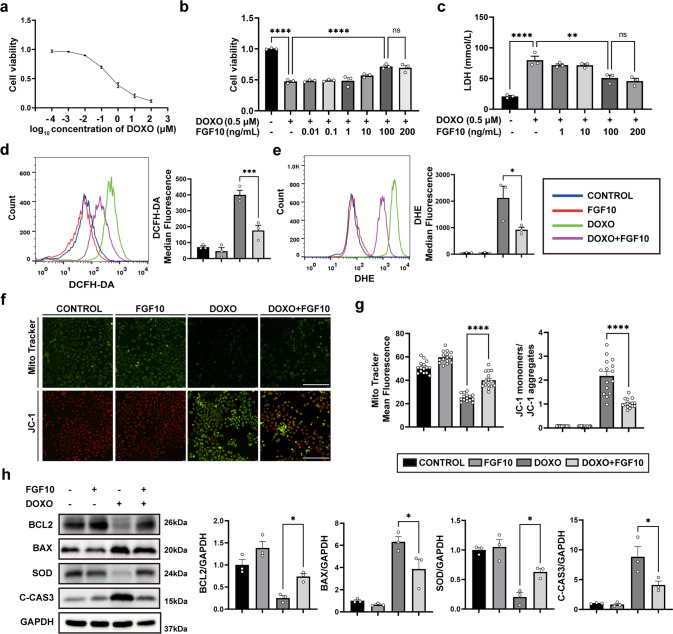
FGF10 mitigates DOXO-induced cytotoxicity in cultured cardiomyocytes. **a** HL-1 cell viability was analyzed using the MTT assay to establish the dose response curve of DOXO at 24 h after DOXO administration. **b** The effect of different concentrations of FGF10 on DOXO treatment was determined. **c** Cell injury was measured by the release of LDH. **d** ROS generation was visualized by DCFH-DA staining and analyzed by flow cytometry. The results are presented as the median fluorescence intensity. **e** Superoxide production was assessed by DHE with flow cytometry. The results are presented as the median fluorescence intensity. **f** Representative images of MitoTracker and JC-1 staining in each group (×400 magnification). **g** Statistical analysis of MitoTracker and JC-1 staining. The results of MitoTracker staining are shown as the mean fluorescence intensity. The results of JC-1 staining are presented as the ratio of the mean fluorescence intensity of JC-1 monomers (green) to the mean fluorescence intensity of JC-1 aggregates (red). **h** Western blotting and statistical analysis of Bcl-2, Bax, SOD, and C-CAS3 expression. GAPDH as an internal control. ns non-significant; *P < 0.05; **P < 0.01; ****P < 0.0001. DCFH-DA Dichlorofluorescin diacetate. Scaler bar: 100 μm.

Having demonstrated the protective role of FGF10 against DOXO-induced myocardial injury, oxidative stress, and apoptosis in vivo, we next investigated the effect of FGF10 on HL-1, a cardiac muscle cell line in vitro. We first tested the dose-dependent cytotoxicity of DOXO in MTT assay (Fig. 4a). Based on this result, we adopted DOXO concentration of 0.5 µM for titration of optimal concentration of FGF10 in reducing the cytotoxicity to 100 ng/mL (Fig. 4b). Additionally, LDH release, which is an indicator of cellular injury, yielded the similar results (Fig. 4c). We therefore choose 0.5 µM of DOXO and 100 ng/mL of FGF10, respectively, as the working concentrations for the further experiments on oxidative stress and apoptosis induced by doxorubicin in HL-1 cells in vitro. DCFH-DA and DHE staining were utilized to measure ROS production, and the results were analyzed using flow cytometry. DOXO increased the median fluorescence intensity of DCFH-DA and DHE, whereas FGF10 substantially decreased the fluorescence intensity when compared to the DOXO group (Fig. 4d, e). Oxidative stress also causes mitochondrial damage and reduces the mitochondrial membrane potential. Therefore, JC-1 and MitoTracker assays were performed to mark mitochondrial oxidative stress. We observed a decrease in MitoTracker fluorescence intensity and a more robust increase in JC-1 monomer green fluorescence intensity. Significantly, FGF10 mitigated the decline in mitochondrial membrane potential induced by DOXO (Fig. 4f, g). Immunoblot analysis revealed drastic reduction of SOD protein expression in DOXO-treated HL-1 cells (Fig. 4h), which was largely prevented by pre-treatment with FGF10. Examination of key apoptosis regulators demonstrated highly increased Bax and C-CAS3 protein expression, and diminished Bcl-2 expression in DOXO group (Fig. 4h), whereas FGF10 treatment significantly mitigated such a pro-apoptotic alteration, further validating the protective effects of FGF10 against doxorubicin-induced cytotoxicity.

The cardioprotective effect of FGF10 was further validated in NRCM. Specifically, our findings showed that FGF10 alleviated DOXO-induced SOD protein reduction in NRCMs (Supplementary Fig. S4c, e). Moreover, the expression level of C-CAS3 was significantly lower in the FGF10 + DOXO group than in the DOXO treatment alone group (Supplementary Fig. S4c, e). Overall, our results suggest that FGF10 displays similar cardioprotective effect in NRCM, which is consistent with results observed in HL-1 (Fig. 4).

### Exogenous FGF10 mitigates DOXO-induced DSBs by improving the NHEJ in vivo and in vitro

**Fig. 5:**
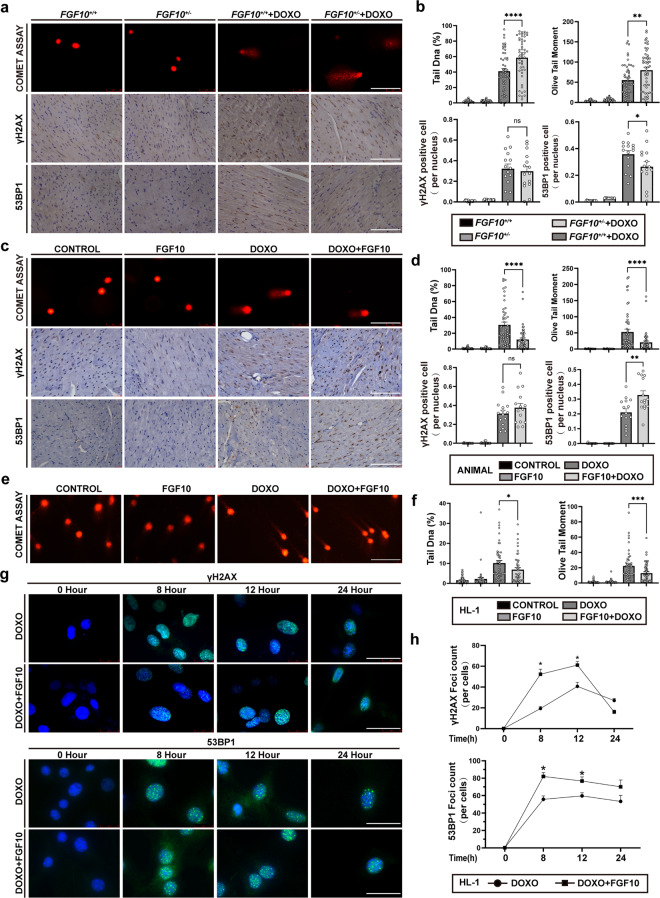
FGF10 mitigates DOXO-induced DSBs and enhances the NHEJ in vivo and in vitro. **a** Representative image of the comet assay and immunohistochemistry of γ-H2AX and 53BP1 in the *Fgf10*^*+/−*^ and *Fgf10*^*+/+*^ mice (×400 magnification). **b** Statistical analysis of the comet assay, γ-H2AX and 53BP1 presented in **a**. The comet assay was used to assess DNA damage through %DNA in tail and tail moment. The results of immunohistochemistry are shown as positive nucleus in all nuclei (per nucleus). **c** Representative image of the comet assay and immunohistochemistry of γ-H2AX and 53BP1 in WT mice from indicated groups (×400 magnification). **d** Statistical analysis of the comet assay, γ-H2AX and 53BP1 presented in **c**. **e** Representative image of the comet assay in HL-1 cells. **f** Statistical analysis of the comet assay in HL-1 cells. **g** Images of γ-H2AX and 53BP1 foci at different time points. **h** Statistical analysis of the number of γ-H2AX and 53BP1 foci. ns non-significant; *P < 0.05; **P < 0.01; ***P < 0.001; ****P < 0.0001. γH2AX gamma-H2A histone family member X, 53BP1 p53-binding protein 1, WT wild type. Scaler bar: 100 μm.

One of the main cytotoxic mechanisms of doxorubicin is mediated by DSBs. To evaluate the potential effect of FGF10 on DOXO-induced DSBs, we first performed a neutral comet assay for DSBs detection in WT (*Fgf10*^*+/+*^) and *Fgf10*^*+/−*^ mice. We used both tail DNA percent and OTM (Olive Tail Moment) to present the result of comet assay. The tail DNA precent is a measure of the relative fluorescent intensity in the head and tail. The OTM is the product of the tail length and the tail DNA precent. Upon DOXO administration, tail DNA percent and OTM were significantly higher in the *Fgf10*^*+/−*^+DOXO group compared with the *Fgf10*^*+/+*^ + DOXO group (*P* < 0.05, Fig. 5a, b). Additionally, the drastic increase of DSBs in DOXO group was significantly diminished in FGF10 pre-treatment group (*P* < 0.0001) (Fig. 5c, d), demonstrating that FGF10 protected against Dox-induced DSBs in vivo.

Homologous recombination (HR) and non-homologous end-joining (NHEJ) are two major DNA repair pathways activated in response to DSBs to maintain cell genomic integrity and stability. The phosphorylation of H2A histone family member X (H2AX) on serine 139 (called γ-H2AX) is the first step in recruiting DNA repair proteins, and γ-H2AX is a sensitive marker of DSB and proxy of DNA damage repair (DDR); whereas p53-binding protein 1 (53BP1) is a key factor for DSB repair in promoting NHEJ. The number of 53BP1 foci is widely used as a measure of NHEJ repair. We therefore examined both γ-H2AX and 53BP1 foci by IHC in DOXO-treated mice. Compared to WT mice, *Fgf10*^*+/−*^ had no significant effect on γ-H2AX expression, but significantly reduced the 53BP1 foci (Fig. 5a, b), whereas pre-treatment with FGF10 highly enhanced DOXO-induced 53BP1 foci formation compared to the DOXO alone group (Fig. 5c, d). Consistent with the above in vivo data, we also observed significant protection of FGF10 against DOXO-induced DSBs in HL-1 cells. Neutral comet assay result showed that DOXO-induced increase in tail DNA and OTM was significantly dampened by FGF10 pre-treatment (Fig. 5e, f). Time kinetic analysis of γ-H2AX and 53BP1 foci demonstrated significant increase in the number of γ-H2AX and 53BP1 foci at 8 h and 12 h in FGF10 + DOXO treatment group compared to DOXO alone group (Fig. 5g, h). Such difference disappeared (γ-H2AX) or reduced (53BP1) between the FGF10 + DOXO and DOXO groups at 24 h, which was likely due to the completion of repair process at this stage. A similar trend was observed in NRCM. The percentage of tail DNA and OTM was significantly decreased after FGF10 treatment (Supplementary Fig. S4a, d). In contrast, 53BP1 foci showed an opposite pattern, with the FGF10 + DOXO group exhibiting more 53BP1 foci than the DOXO group (Supplementary Fig. S4a, d). These results together implicated that FGF10 protected against DSBs by enhancing the NHEJ mechanism of DDR pathway in DOXO-induced myocardial injury.

### The cardioprotective effect of FGF10 is mediated through FGFR2

**Fig. 6:**
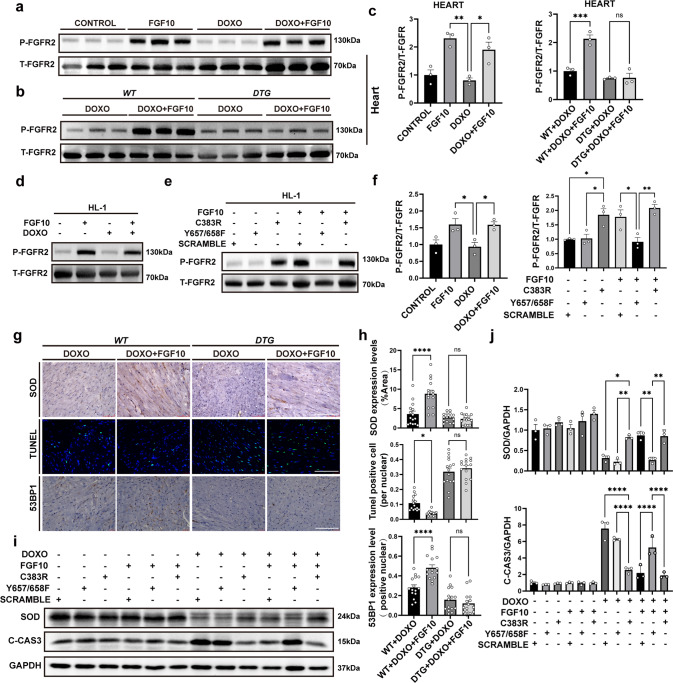
The cardioprotective effect of FGF10 is mediated through FGFR2. **a**, **b** Western blot analyses of FGFR2 and phosphorylated FGFR2 expression in mouse heart tissues of the indicated groups at 12 h post doxorubicin injection. **c** Statistical analysis of the Western blotting data presented in **a** and **b**. **d** Immunoblot detection of FGFR2 and phosphorylated FGFR2 in HL-1 cells of indicated groups at 12 h post doxorubicin administration. **e** Immunoblot detection of total and phospho FGFR2 in FGFR2 stable expression HL-1 stable cells (Vector CONTROL, *C383R* and *Y657/658F, respectively)* with or without FGF10. **f** Statistical analysis of the immunoblot data presented in **d** and **e**. **g** Representative images of SOD, 53BP1, and TUNEL staining in *DTG* and *CTRL* mice. **h** Statistical analysis of the images presented in **g**. **i** Western blot analysis of C-CAS3 and SOD with GAPDH as an internal control in HL-1 cells. **j** Statistical analysis of the data presented in **i**. (*DTG*), *Rosa26*^*rtTA/rtTA*^*; tet(O) sFgfr2b/+*, and the control mice (*CTRL*). ns, non-significant; *P < 0.05; **P < 0.01; ***P < 0.001; ****P < 0.0001. Abbreviations: *DTG*, *Rosa26*^*rtTA/rtTA*^*; tet(O) sFgfr2b/+*; *CTRL*, the control mice. Scaler bar: 100 μm.

Cardiomyocytes both secret FGF10 and express its high affinity receptor FGFR2b [20, 32], therefore FGF10/FGFR2b signaling functions in an autocrine manner in the heart. We found that FGFR2 phosphorylation was highly induced in both FGF10 and FGF10 + DOXO groups at 12 h after DOXO administration in WT mice, HL-1 cells and NRCM (Fig. 6a, c, Supplementary Fig. S4c, e). To elucidate the role of FGFR2 signaling in mediating the protective activity of FGF10, we employed *DTG* mouse model (*Rosa26*^*rtTA*^ */* ^*rtTA*^*; tet(O)sFgfr2b/+*). We found that FGF10-induced FGFR2 phosphorylation was mostly inhibited in doxycycline-treated *DTG* mice compared to *CTRL* (*Rosa26*^*rtTA*^*/* ^*rtTA*^*; +/+*) (Fig. 6b, c) suggesting efficient inhibition of FGFR2 signaling in this system. Importantly, the significant difference in SOD expression, the number of TUNEL-positive cells and the 53BP1 foci between the DOXO + FGF10 and DOXO groups seen in WT mice was eliminated in doxycycline-treated *DTG* mice (Fig. 6g, h). These results demonstrated that FGFR2b activation is of essential importance in mediating the protective effect of FGF10 against oxidative stress, apoptosis, and DSBs. Although FGF10 also induced FGFR2 phosphorylation in HL-1 cells (Fig. 6d, f), the magnitude of activation was not as robust as in the mice (compared to Fig. 6a, b). To further delineate the effect and underpinnings of FGFR2 activation on DOXO-induced cardiotoxicity, we constructed FGFR2 kinase dead (*Y657/658F*) and constitutively active (*C383R*) mutant plasmids based on a lentiviral expression vector, which were used to infect HL-1 cells to generate stable cells. Immunoblot analysis showed that the *C383R* mutant cells displayed high level of phosphorylation even without FGF10 administration. In contrast, FGFR2 phosphorylation remained low in *Y657/658F* mutant cells with or without FGF10 (Fig. 6e, f), confirming that both mutants function in a constitutively active or dominant negative manner as expected. Next we examined the effect of these mutans on DOXO-induced cytotoxicity and FGF10 protection effect in HL-1 stable cells. Compared to the control cell line, the DOXO-induced downregulation of SOD protein expression and concomitant upregulation of C-CAS3 was largely reversed in either *C383R* stable cells or control cells treated with FGF10; whereas the dominant negative *Y657/658F* mutant cells exhibited opposite effect (Fig. 6i, j). These findings indicated that FGF10 exerts its cardioprotective effects in DOXO-induced myocardial injury via FGFR2 activation.

### FGF10 exerts cardioprotective effects via the PHLDA1/Akt axis

**Fig. 7:**
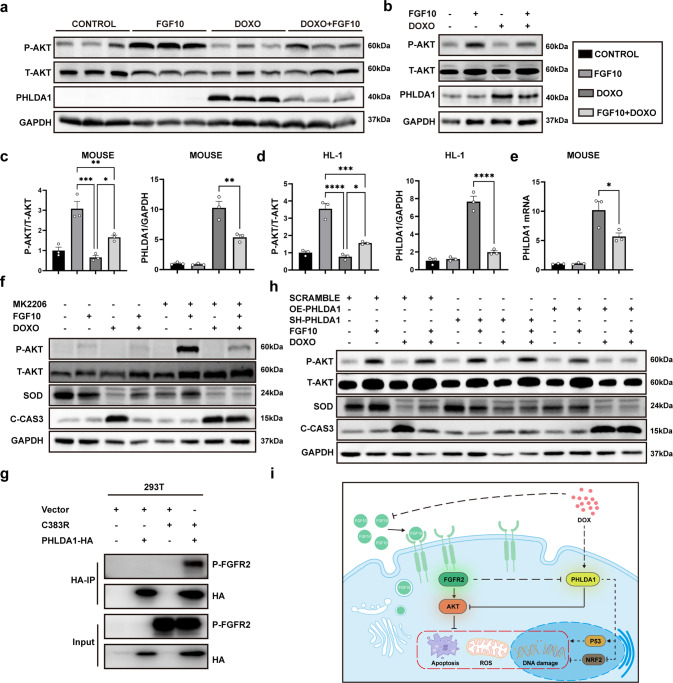
FGF10 exerts cardioprotective effects via the PHLDA1/Akt axis. **a** Western blot analysis of phospho-Akt and total Akt, as well as PHLDA1 in the indicated groups of mice. **b** Western blot analysis of phospho-, total Akt and PHLDA1 in HL-1 cells. **c**, **d** Statistical analysis of the immunoblot data presented in **a** and **b**, respectively. **e** PHLDA1 mRNA expression in mice with RT-qPCR. **f** Western blot analysis of total and phospho-AKT, SOD, and C-CAS3 in HL-1 cells with or without MK2206. **g** Immunoprecipitation against PHLDA1 was performed with pre-conjugated anti-HA agarose beads using 293T protein extracts to determine its interaction with phospho-FGFR2. **h** Western blot analysis of total and phospho-AKT, SOD, and C-CAS3 in HL-1 cells transfected with SH-PHLDA1 or OE-PHLDA1. **i** Cartoon diagram summarizing our findings on FGF10-mediated protection against doxorubicin-induced toxicity. PHLDA1 Pleckstrin homology-like domain family A member 1, ns non-significant; *P < 0.05; **P < 0.01; ***P < 0.001; ****P < 0.0001.

FGF10/FGFR2 signaling mainly leads to activation of the RAS-mitogen-activated protein kinase pathway and the PI3K-Akt pathway. Subsequently, we examined extracellular signal-regulated kinase (ERK) and Akt phosphorylation. Consistent with the previous report [33], we observed that ERK was activated by FGF10 or DOXO administration in cardiac muscle tissue (Supplementary Fig. S5a) and HL-1 cells (Supplementary Fig. S5b). As expected, FGF10 was found to activate Akt both in mouse tissues and in HL-1 and primary cardiomyocyte cultures (Fig. 7a–d, Supplementary Fig. S4c, e). As an important cell survival pathway, Akt activation ameliorates oxidative stress-induced tissue damage and cell apoptosis [34, 35]. To evaluate the impact of Akt activation on FGF10-mediated protective effect, we then examined the effect of blocking the Akt pathway with an Akt inhibitor (MK2206) in HL-1 cells. We observed that MK2206 treatment mostly counteracted the regulatory effect of FGF10 on the induction of SOD expression and inhibition of C-CAS3 expression (Fig. 7f, g). These findings demonstrate that activation of Akt pathway is crucial to the cardioprotective mechanism of FGF10 against DOXO-induced oxidative stress and apoptosis.

Interestingly, although DOXO + FGF10 combined treatment still led to a net increase in Akt phosphorylation compared to non-treat or DOXO alone treated cells, quantitative analysis revealed that the degree of Akt phosphorylation was markedly lower in the DOXO + FGF10 group than in the FGF10 group (*P* < 0.001) as shown in Fig. 7a, c. The data suggests the presence of an additional mechanism of DOXO inhibition of Akt activation, which promoted us to explore other potential factors impacting FGF10/FGFR2/Akt pathway. *PHLDA1* is widely recognized a stress responsive gene, and inhibition of Akt is shared property among the three *PHLDA* family members implicated in suppression of growth stimulatory signaling or promoting apoptosis [36–38]. Interestingly, PHLDA1 is the only member reported to negatively regulate Akt activation in the context of FGFR2 [39]. Strikingly, we found that DOXO administration robustly increased PHLDA1 expression in cardiac tissue (Fig. 7a, c) and HL-1 cells (Fig. 7b, d), as well as primary cardiomyocytes (Supplementary Fig. S4c, e). Importantly, although FGF10 alone exerted no significant effect on PHLDA1 expression, it highly reduced DOXO-induced PHLDA1 expression both in vivo and in vitro (Fig. 7a–d). Consistent with the immunoblot results, quantitative RT-PCR analysis also showed that DOXO-administered mice revealed markedly increased level of *PHDLA1* mRNA expression, which was decreased nearly by half with FGF10 treatment (Fig. 7e). IHC staining of PHLDA1 revealed similar results (Supplementary Fig. S5c). Given the strong DOXO-induced PHLDA1 expression and its correlation with FGF10/FGFR2 signaling mediated protection against DOXO-induced cardiotoxicity, we wanted to further investigate the relationship between FGFR2 and PHLDA1 first by immunofluorescent staining. We found that FGFR2 significantly colocalized with PHLDA1 in cardiac tissue (Supplementary Fig. S6b), NRCM (Supplementary Fig. S4b) and in HL-1 cells (Supplementary Fig. S6c, and Supplementary Table S3, M1 = 0.76 in vitro, M1 = 0.46 in vivo). To address whether phosphorylated FGFR2 may physically interact with PHLDA1, we next performed Co-IP assays. For this purpose, the constitutively active *FGFR2* mutant (C383R) and *PHLDA1-HA* expression plasmids were transfected into HEK293T cells. IP was performed with pre-conjugated anti-HA agarose beads against cell extracts. The result of immunoblot analysis indicated that activated FGFR2 was specifically detected in anti-PHLDA1 precipitates demonstrating a protein complex formation between phospho-FGFR2 and PHLDA1 (Fig. 7h). To directly address the role of PHLDA1 in DOXO-induced cardio cytotoxicity and activated FGF10/FGFR2-mediated protection, we generated three short hairpin RNAs (shRNAs) in pLKO lentiviral vectors to silence PHLDA1, as well as one lentivirus-based expression plasmid (OE-PHLDA1) for stable overexpression in HL-1 cells. The efficiency of PHLDA1 knockdown and overexpression plasmids were validated by immunoblot (Supplementary Fig. S6d). The most effective shRNA (sh-PHLDA1-2) was used for further experiments to delineate the regulatory relationship between FGF10, DOXO and PHLDA1 in HL-1 cells. We found that FGF10 activated the AKT pathway in the control cells as shown in the above-described analyses. However, the inhibition capacity of PHLDA1 disappeared in the sh-PHLDA1 cells. Additionally, FGF10 failed to efficiently activate the AKT pathway after DOXO administration in the OE-PHLDA1 cell (Fig. 7h, Supplementary Fig. S6e).

As demonstrated above, PHLDA1 expression is highly induced by DOXO, which correlated to decreased SOD expression and increased C-CAS3 (Fig. 6i), whereas FGF10 treatment inhibited DOXO-induced PHLDA1 expression (Fig. 7b). Consistently, compared to the control cells, we found that PHLDA1 silenced cells displayed an increase in SOD expression and concomitant decrease in C-CAS3 following DOXO treatment. In contrast, strong pro-apoptotic phenotype was observed in the OE-PHLDA1 cells. However, there was no significant difference between the DOXO alone and DOXO + FGF10 groups in sh-PHLDA1 cells despite the fact that FGF10 downregulated C-CAS3 expression and upregulated SOD expression (Fig. 7h, Supplementary Fig. S6e). Therefore, the protective effects of FGF10 against oxidative stress and apoptosis largely disappeared upon PHLDA1 suppression, demonstrating that PHLDA1 induction plays an important role in regulating Akt activity downstream of FGF10/FGFR2 signaling in the context of DOXO-induced cardiotoxicity.

## Discussion

The cumulative and dose-dependent cardiotoxicity represents the most severe adverse effect of DOXO and the major limiting factor of its clinical efficacy. In the current work, through manipulation of FGF10 expression and FGFR2b activity in genetically engineered mice and in cultured cardiomyocytes, we identify endogenous FGFR2b/PHLDA1/Akt axis as an important molecular underpinning of DOXO-induced cardiotoxicity. We demonstrated that administration of FGF10 delivers robust protection against DOXO-induced oxidative stress, DSBs and apoptosis, which are the main mechanisms underlying its cardiotoxicity. Our main functional and mechanistic findings are summarized in Fig. 7i. We propose that, during DOXO-induced oxidative stress and DSBs leading to pathological damage to cardiac muscle cells and cell death [40], the expression of PHLDA1 is highly induced whereas that of FGFR2b ligands was reduced, both synergistically downregulate Akt activation and contributes to exacerbated DOXO cardiotoxicity and cardiomyocyte death. Administration of exogeneous FGF10 exerts protective effect not only by direct activation of PI3K/Akt via the classical FGF10/FGFR2 signaling, but also indirectly enhancing Akt activity through downregulation of PHLDA1, a potent intrinsic inhibitor of Akt activation. This is, to our knowledge,  it is the first study to unravel the role of the FGF10 in DOXO-induced myocardial injury via this newly identified FGFR2/PHLDA1/Akt pathway.

As an essential morphogen in embryonic development, FGF10 is predominantly expressed in mesenchyme and acts mainly in a paracrine manner to promote epithelial cell proliferation and lineage commitment [20, 32]. Interestingly, a number of studies including ours have documented re-activation of FGF10/FGFR2b signaling in the adult after various injury. Depending on the target tissues or cells and injury models adopted, different mechanisms have been reported to underlie the beneficial activity of FGF10. In epithelial targeted injury models, overexpression of FGF10 via adeno-associated virus, transgenics expression or administration of FGF10 promotes epithelial repair/regeneration and inhibits apoptosis, such as the BLM-induced lung injury [15, 17, 41, 42], ischemia reperfusion (I/R) injury to the kidney [13, 43] or liver [12]. FGF10 also functions in an autocrine manner in the heart to promote adult cardiac tissue repair and regeneration, or endothelial cell proliferation [20, 22, 23]. Hubert et al shows that endogenous Fgf10 expression is up-regulated in the injured ventricle after ischemic myocardial infarction (MI). Fgf10 hypomorph mice exhibit reduced cardiomyocyte proliferation and enhanced cardiac fibrosis, whereas induced Fgf10 overexpression enhances post MI cardiomyocyte proliferation and prevents myofibroblast activation, and better preserves cardiac function [23].

Thus far, FGF10 has been shown to modulate various key cellular processes and activities, such as apoptosis, cell proliferation, autophagy, ER stress, fibrosis, inflammation, etc. Interestingly, PI3K/Akt activation has emerged as an important pathway mediating the protective effect of FGF10 in the liver, lung and neuronal tissues, as well as in the heart [12, 13, 22, 42]. Therefore, understanding how endogenous PI3K/Akt is regulated under various injury conditions and to harness the reparative capacity of FGF10 and target this key survival pathway is of crucial importance.

Along this line, increased FGF10 expression and activation of FGFR2 signaling is reported in liver and kidney exposed to I/R injury [12, 13, 43]. Upregulation of FGF10 is also observed in ischemic MI, and is suggested to be driven by reactivation of the embryonic transcriptional program [23]. In contrast, we found that the expression of endogenous FGF10 decreased in cardiac tissue along with other FGFR2b ligands following DOXO exposure. Additionally, we noticed that the degree of Akt phosphorylation was lower in FGF10 + DOXO group compared to FGF10 treatment alone group suggesting additional DOXO-related mechanism might exist to suppress Akt activation in the heart.

Interestingly, in a microarray profiling for transcriptional target of FGFR inhibitor (FGFRi) in endometrial cancer cells, PHLDA1 was identified as the most significantly downregulated gene in the resistant cells [39, 44]. PHLDA1 negatively regulates Akt in the context of FGFR2, whereas loss of PHLDA1 expression contributes to cancer cell survival and FGFRi resistance due to reactivation of Akt [39, 44]. Therefore, we undertook the initiative to examine PHLDA1 expression in DOXO-treated mice, which not only demonstrated its DOXO-induced expression, but also mutual regulation of FGF10/FGFR2 and PHLDA1 on Akt activation. From our perspective, PHLDA1 acts as an acute response protein in response to DOXO-injury to promote apoptosis through inhibition of Akt, which is consistent with several previous studies, such as elevated expression of PHLDA1 in rat ischemic cardiomyopathy model and in H9C2 cells cultured under hypoxia condition [45]. PHLDA1 overexpression promotes H9C2 cell apoptosis, which is associated with down-regulation of Akt phosphorylation. While our work is ongoing, another study reported PHLDA1 expression was induced following myocardial I/R-injury, and adenovirus-mediated shRNA knockdown of PHLDA1 significantly alleviated MI size and cardiomyocyte apoptosis [46].

Inhibition of Akt activation is a key activity of PHLDA1 conserved in all three members of this family [36–39]. PHLDA1 localizes to the plasma membrane through the pleckstrin-homology domain, which competes with Akt in binding to phosphatidylinositol phosphate (PIP) 3 or PIP2 to represses Akt activation [38]. Knockdown of PHLDA1 in different types of cancer cells promotes Akt activation, whereas overexpression of PHLDA1 reduced its activation [38]. Functionally, suppression of PHLDA1 mRNA expression was sufficient to reduce high-glucose stimulated apoptosis, oxidative stress, and inflammation in kidney podocytes, via Nrf2 activation [47]. Therefore, a robust induction of PHLDA1 in cardiomyocytes likely plays a prominent role in promoting DOXO-induced cardiotoxicity through inhibition of Akt.

Our data not only show that PHLDA1 colocalizes with phosphorylated FGFR2 in cardiac cells in mice and HL-1 cells (Supplementary Fig. S6b, c), but also confirmed their physical interactions by co-IP experiment (Fig. 7g). Further experiments through the manipulation of PHLDA1 expression in HL-1 cells with dominant negative or constitutively active FGFR2b mutant, respectively, confirm FGFR2b as a predominant FGF10 receptor upstream of DOXO-induced PHLDA1/Akt activity, despite that FGFR1b is also reported to be expressed in adult heart. Of note, apart from Akt inhibition, other proapoptotic mechanisms have also been reported for PHLDA1. PHLDA1inhibits ErbB receptor, particularly ERBB2,3 oligomerization, to suppress its down-stream signaling [48], and also modulates the cell susceptibility to apoptosis via the endoplasmic reticulum stress response pathway in the cancer setting [49]. PHLDA1 has been identified as a transcriptional target of p53 by various types of DNA damage [38]. It will be interesting to investigate whether similar mechanism exists in DOXO-induced PHLDA1 in cardiomyocytes in the future.

We demonstrated in cardiac tissues by neutral comet assay that DSBs is the primary form of DOXO-induced damage. DSBs are highly deleterious for cell viability, and if not repaired, cause apoptosis. Although considered as an error-free repair system, HR mechanism is restricted to the S-G2 phases of the cell cycle [50]. Since the adult mammalian cardiomyocytes have exited the cell cycle with extremely limited potential of proliferation, the repair of DSBs in cardiomyocytes cannot be achieved through HR [51]. Therefore, by far the majority of DSBs in cardiomyocytes are repaired though NHEJ. Despite being a less accurate and mutagenic repair system, NHEJ can occur throughout the cell cycle and has an impotent protective role in maintaining genome integrity and stability. In our study, we detected a decrease in DSBs and an increase in NHEJ activity following FGF10 treatment in DOXO-induced myocardial injury. Augmented DNA repair induced by H_2_O_2_ was also found in the lungs following treatment with FGF10 [52]. More recent studies have shown that FGF10 pre-treatment protected immortalized human keratinocytes (HaCaT) and human epidermal equivalents from ultraviolet B-induced DSBs based on measurement of γ-H2AX expression and cyclobutane pyrimidine dimers. At cellular level, FGF10 attenuates ROS production, apoptosis, DNA damage, and mitochondrial dysfunction caused by UVB exposure, which is suggested to be mediated by Nrf2 pathway triggered by the aryl hydrocarbon receptor (AhR) activation [53]; Similar protection against UVB was also reported in mouse skin via an ERK/Yes-Associated Protein (YAP)-dependent pathway [54]. Increased NHEJ activity aids cardiomyocyte survival, but misrepair can occur, which leads to increased risk of oncogenic transformation [55], but this is not considered a significant concern for cardiomyocytes given that they exit cell cycle and the treatment duration of FGF10 is temporary. Therefore, FGF10 may offer an alternative management option for DOXO-induced DSBs.

In conclusion, our findings reveal that DOXO-induced cardiotoxicity is underpinned by its down-regulation of intrinsic FGFR2b ligand expression along with a robust induction of pro-apoptotic protein PHLDA1, both act in synergy to inhibit Akt pathway compromising cardiomyocyte survival in response to the injury. FGF10 treatment ameliorates DOXO-induced oxidative stress, DSBs and improves NHEJ repair to preserve cardiac function. Mechanistically, we identify FGFR2/PHLDA1/Akt axis as a predominant pathway in FGF10-mediated protection against DOXO induced cardiomyocyte toxicity in vivo and in vitro. Together, our results support FGF10 as a potential cardioprotective agent for patients receiving DOXO therapy to prevent cardiovascular complications.

### Supplementary information

Supplement file

Supplementary Figure S1

Supplementary Figure S2

Supplementary Figure S3

Supplementary Figure S4

Supplementary Figure S5

Supplementary Figure S6
