# Supplementary material for: FGF10 mitigates doxorubicin-induced myocardial toxicity in mice via activation of FGFR2b/PHLDA1/AKT axis
Source: Acta Pharmacol Sin. 2023 May 24;44(10):2004–18. doi: 10.1038/s41401-023-01101-x (PMC10545682; doi:10.1038/s41401-023-01101-x)
Supplement: Supplementary file 1 — Supplement file [file 41401_2023_1101_MOESM1_ESM.docx]

**Supplementary FILES**

**Figure S1.** **(a)** Representative image of agarose gel electrophoresis for *Fgf10^+/−^* genotyping. **(b)** Representative gel electrophoresis images of *Rosa26^rtTA/rtTA^; tet(O)sFgfr2b/+* genotyping. Left panel: *Rosa26^rtTA/rtTA^* genotyping PCR; Right panel: *sFgfr2b/+* genotyping PCR*.* **(c)** Immunoblot analysis of FGF10 in the *Fgf10^+/−^* and *Fgf10^+/+^* mice. **(d)** Statistical analysis of the immunoblot data presented in **(a).** ***P* < 0.01.

**Figure S2. (a)** Schematic of the experimental timeline for *Fgf10^+/−^* and *Fgf10^+/+^* mice. **(b)** Schematic of the experimental timelines for FGF10 and/or DOXO treatment in HL-1 cell. **(c)** Schematic of the experimental timelines for FGF10 and/or DOXO treatment in C57BL/6 mice. **(d)** Schematic of the experimental timelines for FGF10 and/or DOXO treatment in *CTRL* and *DTG* mice. Abbreviations: *CTRL, Rosa26^rtTA/rtTA^; +/+*; *DTG, Rosa26^rtTA/rtTA^; tet(O)sFgfr2b/+.*

**Figure S3. (a)** Representative echocardiography images in the *Fgf10^+/+^* and *Fgf10^+/−^* mice after DOXO injection. **(b)** Representative H&E staining images in the *Fgf10^+/+^* and *Fgf10^+/−^* mice. **(c)** Representative echocardiography images in each group after DOXO injection. **(d)** Representative H&E staining images in each group. Scale bar: 100 μm.

**Figure S4. (a)** Representative images of the comet assays and immunofluorescence of 53BP1 in indicated groups of NRCM. **(b)** Representative images of PHLDA1 and FGFR2 (*C383R*) colocalization in NRCM. **(c)** Western blot analysis of total and phospho-FGFR2, total and phospho-AKT, SOD, C-CAS3 and PHLDA1 in NRCM. **(d)** Statistical analysis of the comet assays and 53BP1 staining presented in **(a). (e)** Statistical analysis of the Western blot presented in **(c).** **P* < 0.05; ***P* < 0.01; ****P* < 0.001; *****P* < 0.0001. Abbreviations: NRCM, Neonatal Rat Cardiomyocyte.

**Figure S5. (a)** Western blot analysis of total and phosphor-ERK in mouse heart. **(b)** Western blot analysis of total and phosphor-ERK in HL-1 cell. **(c)** Representative image of PHLDA1 immunohistochemical staining in the heart of each group.

**Figure S6.** (a) Quantification and statistical analysis of immunoblot results for phospho-Akt, AKT, SOD and C-CAS3 shown in Fig. 7f. **(b)** Representative images of PHLDA1/FGFR2 colocalization in mouse heart tissues. DAPI (Blue), PHLDA1 (Red), P-FGFR2 (Green). **(c)** HL-1 cells were transfected with FGFR2 (*C383R* mutant) expression plasmid and immunofluorescent stained for confocal imaging. The vector contains internal GFP expression cassette controlled by a separate EF-1a promoter to mark the transfected cells. FGFR2-Flag protein (Grey). DAPI (Blue), PHLDA1 (Red), GFP (Green). **(d)** Immunoblot confirmation of PHLDA1 expression in PHLDA1 stable knockdown and PHLDA1 stable overexpression HL-1 cells. **(e)** Quantification and statistical analysis of immunoblot results for phosphor-Akt, AKT, SOD and C-CAS3 shown in Fig. 7h.

**Table S1.** List of primers used in article.

| Primer | Sequence |
| --- | --- |
| *FGF3*-F | 5’-TGCGCTACCAAGTACCACC-3’ |
| *FGF3*-R | 5’-CACTTCCACCGCAGTAATCTC-3’ |
| *FGF7*-F | 5’-CTCTACAGGTCATGCTTCCACC-3’ |
| *FGF7*-R | 5’-ACAGAACAGTCTTCTCACCCT-3’ |
| *FGF10*-F | 5’-TTTGGTGTCTTCGTTCCCTGT-3’ |
| *FGF10*-R | 5’-TAGCTCCGCACATGCCTTC-3’ |
| *FGF22*-F | 5’-CCAGGACAGTATAGTGGAGATCC-3’ |
| *FGF22*-R | 5’-AGTAGACCCGCGACCCATAG-3’ |
| *CAT*-F | 5’-AGCGACCAGATGAAGCAGTG-3’ |
| *CAT*-R | 5’-TCCGCTCTCTGTCAAAGTGTG-3’ |
| *PRDX1*-F | 5’-AATGCAAAAATTGGGTATCCTGC-3’ |
| *PRDX1*-R | 5’-CGTGGGACACACAAAAGTAAAGT-3’ |
| *GPX1*-F | 5’-AGTCCACCGTGTATGCCTTCT-3’ |
| *GPX1*-R | 5’-GAGACGCGACATTCTCAATGA-3’ |
| *PHLDA1*-F | 5’-GGGCTACTGCTCATACCGC-3’ |
| *PHLDA1*-R | 5’-AAAAGTGCAATTCCTTCAGCTTG-3’ |
| rtTA Flox 5’ | 5’-GAGTTCTCTGCTGCCTCCTG-3’ |
| rtTA Flox 3’ | 5’-CGAGGCGGATACAAGCAATA-3’ |
| rtTA Flox Jody 3’ | 5’-AAGACCGCGAAGAGTTTGTC-3’ |
| SolFgfr2b-F | 5’-GAAGGAGATCACGGCTTCC-3’ |
| SolFgfr2b-R | 5’-AGACAGATGATACTTCTGGGACTGT-3’ |
| Fgf10 WT-F | 5’-CATTGTGCCTCAGCCTTTCC-3’ |
| Fgf10 MT-F | 5’-CACCAAAGAACGGAGCCGGTTG-3’ |
| Fgf10 COM | 5’-GGCCACATCTGGAACAGATT-3’ |
| *ACTB*-F | 5’-GGCTGTATTCCCCTCCATCG-3’ |
| *ACTB*-R | 5’-CCAGTTGGTAACAATGCCATGT-3’ |
| shPHLDA1-1 | 5’-GGGCAAAGAGATCGACTTTCG-3’ |
| shPHLDA1-2 | 5’-GCTACTGCTCATACCGCCCAA-3’ |
| shPHLDA1-3 | 5’-GCGTCGAGATGACTTTATTTA-3’ |

**Table S2.** List of primary antibodies used in article.

| Antibody |  |
| --- | --- |
| 53BP1 | Abcam (ab175933) |
| Gamma-H2AX | Abcam (ab81299) |
| FGFR2 | Abcam (ab289968) |
| PHLDA1 | Abcam (ab133654) |
| Anti-HA tag Agarose | Abcam (ab32042) |
| Cleaved Caspase-3 | Abcam (ab32042) |
| BAX | Cell Signaling Technology (89477) |
| Phospho-AKT (Ser473) | Cell Signaling Technology (4060) |
| Akt (pan) (11E7) | Cell Signaling Technology (4685) |
| Phospho-p44/42 MAPK (Erk1/2) | Cell Signaling Technology (4370) |
| p44/42 MAPK (Erk1/2) | Cell Signaling Technology (4695) |
| SOD2 | Santa Cruz (sc-137254) |
| BCL2 | Santa Cruz (sc-7382) |
| PHLDA1 | Santa Cruz (sc-23866) |
| GPX4 | Proteintech (Cat No. 67763-1-Ig) |
| CAT | Proteintech (Cat No. 21260-1-AP) |
| FGF10 | Sigma-Aldrich (ABN44) |
| Phospho-FGFR2 (Ser782) | Thermo Fisher (PA5-106140) |
| GAPDH | Bioworlde (MB001H) |

**Table S3.** Colocalization analyze of PHLDA1 and p-FGFR2^*^.

|  | *R^#^* | M1^$^ | M2^&^ |
| --- | --- | --- | --- |
| Cell | 0.28±0.13 | 0.76±0.21 | 0.39±0.14 |
| Animal | 0.50±0.08 | 0.46±0.07 | 0.44±0.07 |

^*^ HL-1 cells were transfected with FGFR2 (C383R mutant) expression plasmid and immunofluorescent stained for confocal imaging. The vector contains internal GFP expression cassette controlled by a separate EF-1a promoter to mark the transfected cells. FGFR2-Flag protein (Grey). DAPI (Blue), PHLDA1 (Red), GFP (Green).

^#^ Analyze by person correlation.

^$^ Analyze by Manders’ correlation. Faction of FGFR2 overlapping PHLDA1 in animal, while faction of Flag overlapping PHLDA1 in cell.

^&^ Analyze by Manders’ correlation. Faction of PHLDA1 overlapping FGFR2 in animal, while faction of PHLDA1 overlapping Flag in cell.
