# Supplementary figures and images for: FGF10 mitigates doxorubicin-induced myocardial toxicity in mice via activation of FGFR2b/PHLDA1/AKT axis
Source: Acta Pharmacol Sin. 2023 May 24;44(10):2004–18. doi: 10.1038/s41401-023-01101-x (PMC10545682; doi:10.1038/s41401-023-01101-x)

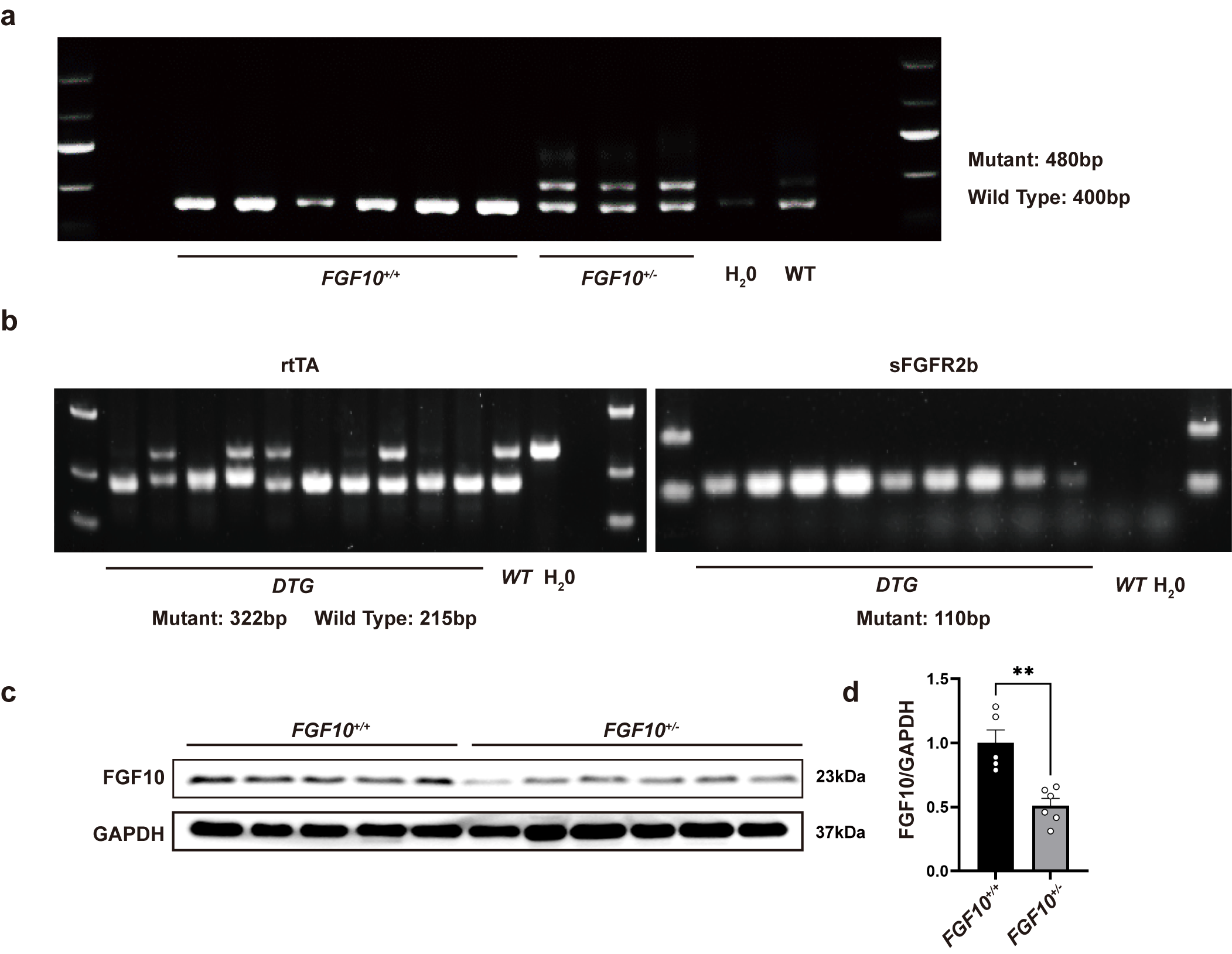

Supplement: Supplementary file 2 — Supplementary Figure S1 [file 41401_2023_1101_MOESM2_ESM.tif]

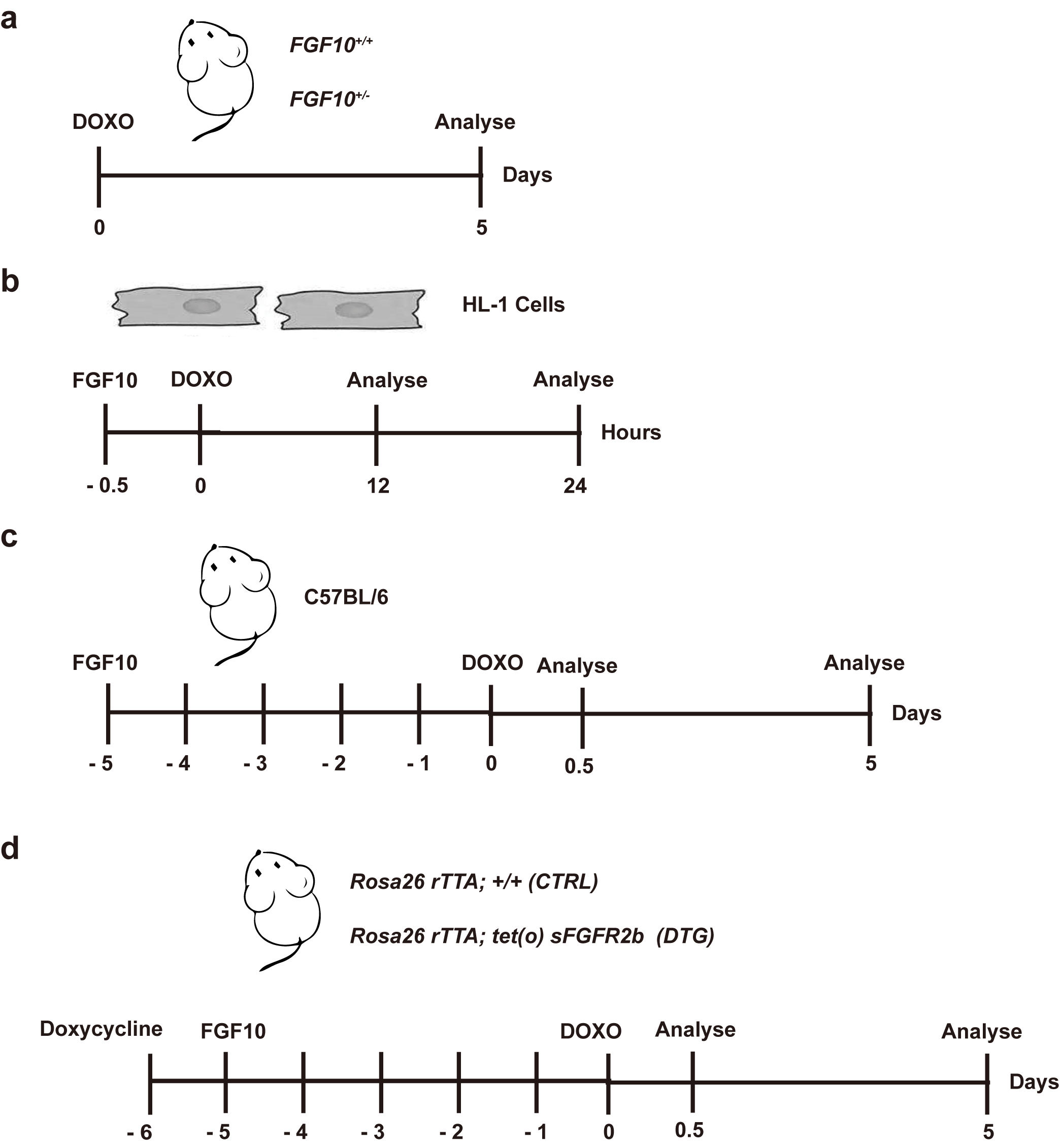

Supplement: Supplementary file 3 — Supplementary Figure S2 [file 41401_2023_1101_MOESM3_ESM.tif]

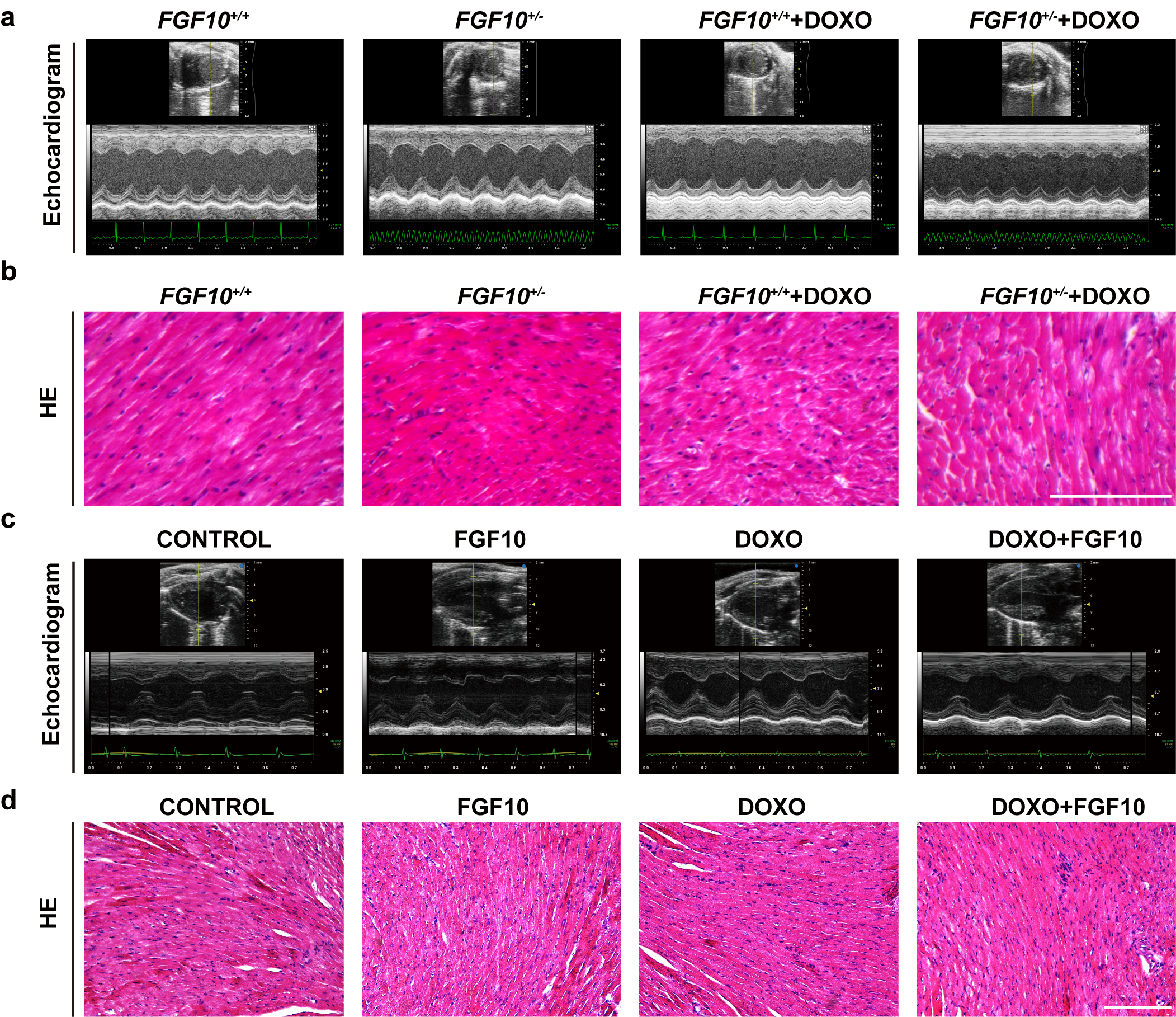

Supplement: Supplementary file 4 — Supplementary Figure S3 [file 41401_2023_1101_MOESM4_ESM.tif]

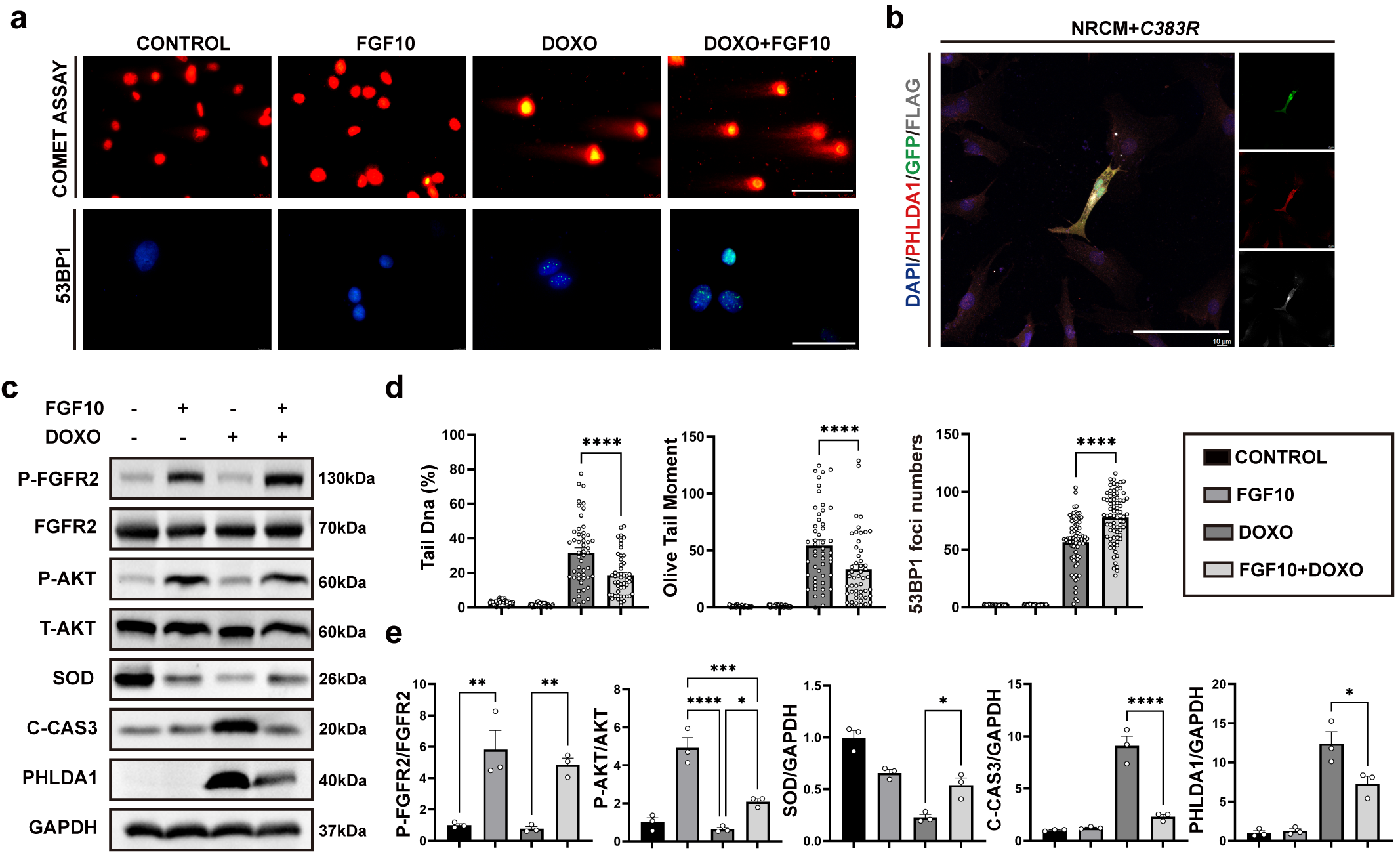

Supplement: Supplementary file 5 — Supplementary Figure S4 [file 41401_2023_1101_MOESM5_ESM.tif]

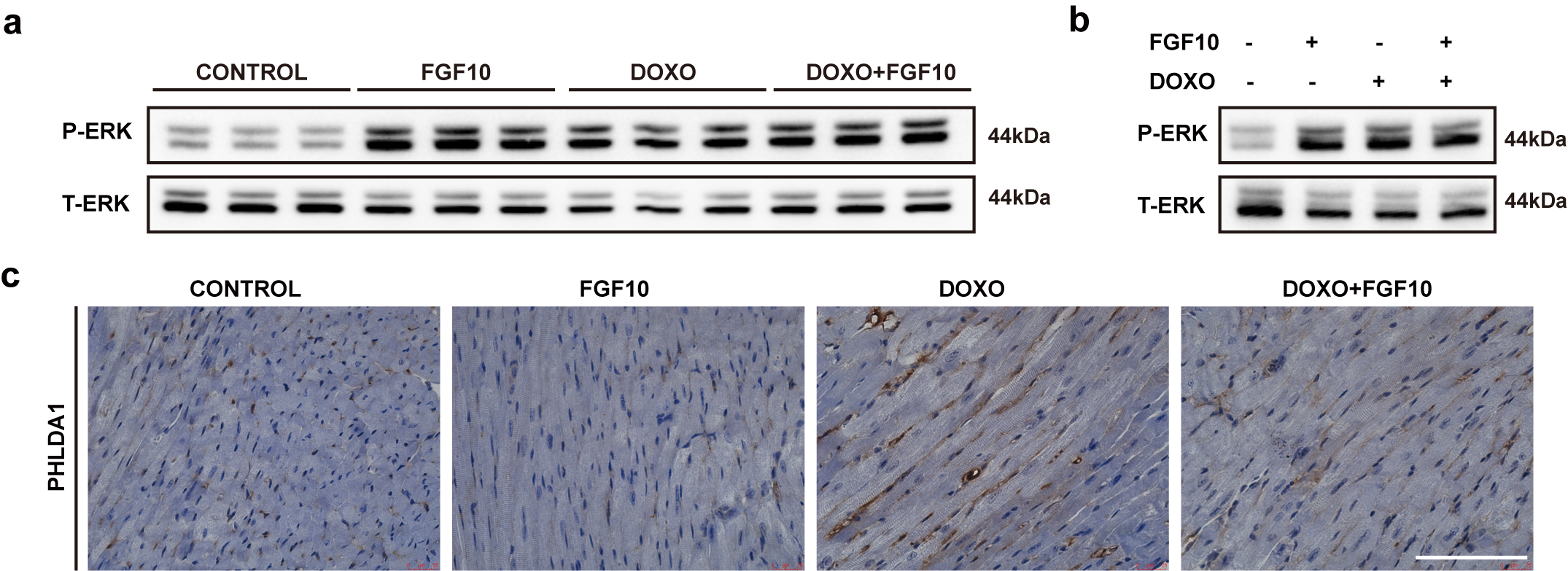

Supplement: Supplementary file 6 — Supplementary Figure S5 [file 41401_2023_1101_MOESM6_ESM.tif]

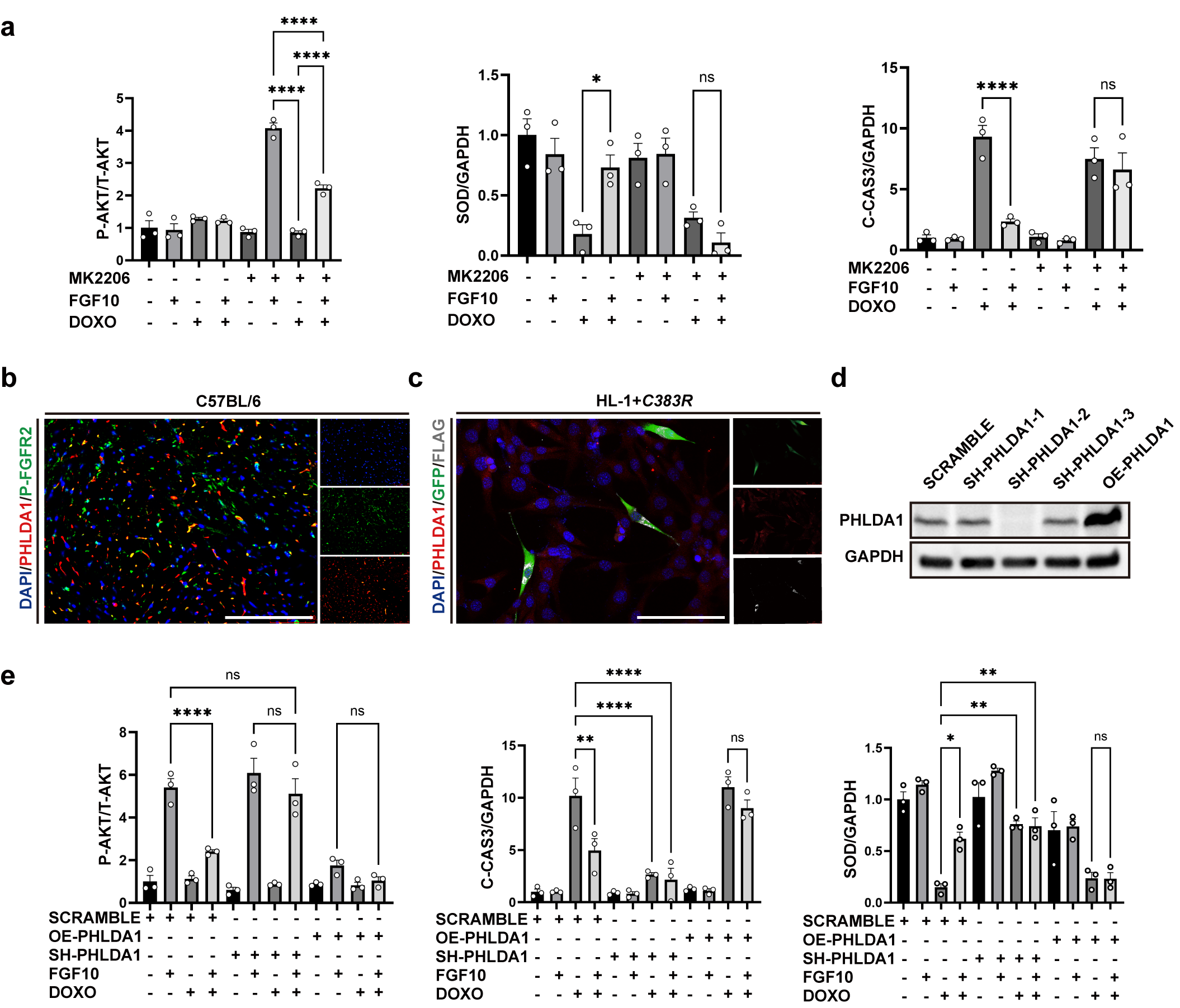

Supplement: Supplementary file 7 — Supplementary Figure S6 [file 41401_2023_1101_MOESM7_ESM.tif]
